# Characterization of Chronic Rhinosinusitis Patients Based on Markers of Type 2 Inflammation: Findings From the European CRS Outcome Registry (CHRINOSOR)

**DOI:** 10.1002/clt2.70095

**Published:** 2025-08-31

**Authors:** Carlo Cavaliere, Sven F. Seys, Joost de Kinderen, Giulia Bettio, Alexandros Andrianakis, Isam Alobid, Peter W. Hellings, Laura Van Gerven, Valérie Hox, Claire Hopkins, Anette Kjeldsen, Sietze Reitsma, Sven Schneider, Peter‐Valentin Tomazic, Zuzana Diamant, Julia Eckl‐Dorna, Wytske J. Fokkens, Clemens Holzmeister, Kenneth Larsen, Anu Laulajainen‐Hongisto, Antonella Loperfido, Valerie Lund, Gert Mariën, Simonetta Masieri, Geoffrey Mortuaire, Mathilde Moyaert, Joaquim Mullol, Josje Otten, Camilo Rodriquez‐Van Strahlen, Martin Wagenmann, Claus Bachert

**Affiliations:** ^1^ Department of Sense Organs Sapienza University Rome Italy; ^2^ Galenus Health Hasselt Belgium; ^3^ Department of General ORL, Head and Neck Surgery Medical University of Graz Graz Austria; ^4^ Rhinology and Skull Base Surgery Unit Otorhinolaryngology Department Hospital Clinic IDIBAPS Ciberes Universitat de Barcelona Barcelona Spain; ^5^ Department of Otorhinolaryngology‐Head and Neck Surgery UZ Leuven Leuven Belgium; ^6^ Allergy and Clinical Immunology Research Group Department of Microbiology, Immunology & Transplantation KU Leuven Leuven Belgium; ^7^ Department of Neurosciences Experimental Otorhinolaryngology Rhinology Research KU Leuven Leuven Belgium; ^8^ Service d’Otorhinolaryngologie Cliniques Universitaires Saint‐Luc Brussels Belgium; ^9^ ENT Department Guy's and St Thomas' NHS Foundation Trust London UK; ^10^ Department of Otorhinolaryngology Odense University Hospital Odense Denmark; ^11^ Department of Otorhinolaryngology and Head/neck Surgery Amsterdam University Medical Centre University of Amsterdam Amsterdam the Netherlands; ^12^ Department of Otorhinolaryngology, Head and Neck Surgery Vienna General Hospital (AKH) Medical University of Vienna Vienna Austria; ^13^ Department of Respiratory Medicine First Faculty of Medicine Charles University and Thomayer Hospital Prague Czech Republic; ^14^ Department of Clinical Pharmacology and Pharmacology University of Groningen University Medical Center Groningen Groningen the Netherlands; ^15^ Department of Otorhinolaryngology‐Head and Neck Surgery Helsinki University Hospital and University of Helsinki Helsinki Finland; ^16^ Otolaryngology Unit San Camillo Forlanini Hospital Rome Italy; ^17^ Department of Rhinology Royal National Ear, Nose, Throat and Eastman Dental Hospital London UK; ^18^ Department of Oral and Maxillofacial Sciences Sapienza University Rome Italy; ^19^ Otorhinolaryngology‐Head and Neck Department Huriez Hospital Centre Hospitalier Universitaire (CHU) Lille Lille France; ^20^ Department of Otorhinolaryngology Universitätsklinikum Düsseldorf (UKD) Dusseldorf Germany; ^21^ Clinic for ENT Diseases and Head and Neck Surgery University Clinic Münster Münster Germany; ^22^ Department of Otorhinolaryngology First Affiliated Hospital Sun Yat‐Sen University International Airway Research Center Guangzhou China

**Keywords:** asthma, eosinophils, mobile application, real world data, total IgE

## Abstract

**Background:**

Primary chronic rhinosinusitis (CRS) can be classified based on the sinuses involved and the dominant endotype of the mucosal inflammation. Since the introduction of type 2 targeted biologics as treatment option for CRS, assessment of the inflammatory status has gained importance in CRS patients. We here aimed to characterize CRS patients with and without elevated markers of type 2 inflammation.

**Methods:**

CRS patients who visited the outpatient ENT clinic in one of the 10 tertiary centers in 7 European countries were invited to use the Galenus Health mobile application for the monitoring of their disease.

**Results:**

CRS patients (*n* = 281) were stratified according to blood eosinophil counts or BEC (< 150 cells/μL: 21.6% of patients, ≥ 150 cells/μL: 78.4%; < 250 cells/μL: 36.3%, ≥ 250 cells/μL: 63.7%) and serum total IgE (< 100 IU/mL: 59.9%, ≥ 100 IU/mL: 40.1%). BEC and serum total IgE did not correlate well (Spearman *r* = 0.06; *p* = 0.39). CRS patients with BEC ≥ 150 cell/μL or ≥ 250 cells/μL, respectively, showed increased NPS, SNOT‐22, VAS for total CRS symptoms, loss of smell, nasal blockage, runny nose compared to patients with BEC below 150 or 250 cells/μL. CRS patients with increased serum total IgE (≥ 100 IU/mL) did not show differences in the outcome parameters compared to patients with levels below 100 IU/mL. CRS patients with asthma (58.9%) showed increased SNOT‐22 and VAS loss of smell compared to patients without asthma.

**Conclusions:**

A significant proportion of CRS patients exhibit a type 2 endotype, characterized by blood eosinophilia (78%), increased serum total IgE (40%) and/or concomitant asthma (59%). Our results underline the usefulness of eosinophils as a marker of type 2 inflammation and severity but challenge the utility of serum total IgE since it does not correlate with any of the markers of severity.

## 1 Introduction

Chronic rhinosinusitis (CRS) is an inflammatory condition of the nasal and paranasal sinus mucosa that affects 5%–12% of the worldwide population [1, 2]. This chronic condition is associated with substantial morbidity and a marked reduction in health‐related quality of life (HRQoL), affecting patients’ overall well‐being, compromising daily functioning as well as sleep quality [3]. Saline irrigations, intranasal and systemic corticosteroids, along with endoscopic sinus surgery (ESS), have been the mainstay in the therapeutic management of CRS for several decades [4]. In particular, for patients with CRS with nasal polyps (CRSwNP), it has been demonstrated in recent years that appropriate pharmacotherapy when combined with ESS, is significantly more efficient than pharmacotherapy alone [5]. However, despite appropriate pharmacological and surgical treatments, lack of control of the disease is frequent [6, 7, 8]. Therefore, a more detailed analysis of CRS patients with uncontrolled disease in real‐world clinical settings is essential to gain a deeper understanding of the predictive factors and causes of uncontrolled disease or disease persistence [9]. In this regard, it has been reported before that type 2 inflammation represents the predominant endotype of uncontrolled CRSwNP, characterized by a significantly elevated presence of mast cells, eosinophils and basophils, as well as high levels of IgE, Th2 cells, and type 2 cytokines (IL‐4, IL‐5, and IL‐13) in Western countries [10, 11].

In clinical practice, patients with CRSwNP associated with type 2 inflammation typically present with increased blood and/or tissue eosinophils, elevated IgE levels and bilateral nasal polyps as well as with thick nasal mucus (allergic/eosinophilic mucin) observed at nasal endoscopy [12]. These patients are often prescribed systemic corticosteroids (SCS), typically have a history of revision ESS and experience a significant reduction in QoL, particularly in their sense of smell, as they are commonly hyposmic or anosmic [13]. Additionally, they frequently present with type 2 comorbidities, including non‐steroidal drug‐exacerbated respiratory disease (NERD), bronchial asthma, atopic dermatitis, allergic rhinitis, and eosinophilic esophagitis [14, 15].

More recently, biologics targeting type 2 inflammatory pathways, such as dupilumab, mepolizumab, and omalizumab, have received regulatory approval for the treatment of uncontrolled severe CRSwNP [16, 17]. More specifically, dupilumab targets IL‐4 receptor α (IL‐4Rα), the common chain of IL‐4 and IL‐13 receptors and consequently inhibits IL‐4 and IL‐13 signaling [18], omalizumab is an anti‐IgE antibody [19] and mepolizumab inhibits IL‐5 signaling [20]. These therapies have become widely integrated into routine clinical practice in many countries, offering a significant advancement in the management of this condition [21, 22, 23]. However, the clinical outcome of biologic therapies demonstrates considerable heterogeneity among patients [24].

A comprehensive and detailed analysis of clinical parameters, including treatable traits and comorbidities, along with the evaluation of relevant biological markers, is therefore essential to optimize the selection of the most appropriate biologic treatment for each individual patient [25]. Furthermore, in this context, real‐world data plays a crucial role in enhancing our knowledge within this field and beyond [26, 27, 28].

To that end, a consortium comprised of European CRS scientific experts and Galenus Health, a digital platform providing research support and digital technology, have collaborated to create an international CHRonic rhINOSinusitis Outcome Registry (CHRINOSOR) [29]. To date, this international registry comprises 10 tertiary referral centers across Europe and is dedicated to the collection and analysis of real‐world data from adult patients diagnosed with CRS. The objective of the current study was to characterize the primary features of CRS patients within CHRINOSOR, and more specifically, patients with and without type 2 inflammation.

## 2 Methods

### 2.1 Study Design and Population

This observational, multicentre study was conducted in 10 centers from 7 European countries: Austria, Belgium, Denmark, Italy, Spain, The Netherlands, and the United Kingdom. Data was collected to establish a cohort of prospectively recruited CRS patients who visited the outpatient clinic of the participating tertiary centers in a real‐world setting. Participating patients were invited to use the Galenus Health app [29] for the follow‐up of their condition. The study was approved by the local institutional review boards and written informed consent was obtained, except for The Netherlands (not required according to the Dutch Medical Research Involving Human Subject Act) and Denmark (not required according to national legislation). The study was registered at clinicaltrials.gov (NCT04670172).

### 2.2 Inclusion and Exclusion Criteria

Adult patients with CRS diagnosis according to EPOS 2020 were included in this cohort. In order to reflect the real‐life CRS population as much as possible, the number of exclusion criteria were kept to a minimum. Only patients presenting with malignancies, inverted papilloma or unilateral disease were excluded.

### 2.3 Data Collection and Outcome Measures

Patients were invited to complete their health profile at the start of the study using the Galenus Health app. As previously described [29], the health profile consists of an electronic questionnaire to capture the patient's demographic characteristics (age, gender, body mass index [BMI], smoking history), disease history (number of courses of systemic corticosteroids [SCS] in the past year, number of endoscopic sinus surgeries [ESS] in the past year), presence of comorbidities (NSAID exacerbated respiratory disease [NERD], asthma, allergy). Patients were invited on a weekly basis to complete the health diary and to collect information on total and specific CRS‐related symptoms as well as impact on daily life activities and sleep disturbance by a visual analog scale (VAS) (ranging from 0 “no burdensome symptoms” to 100 “extremely burdensome symptoms”). Disease‐specific, health‐related questionnaires, Sino‐Nasal Outcome Test‐22 (SNOT‐22; on a total score of 110) and Asthma Control Test (ACT; on total score of 25), were completed at the start of using the app and patients received a monthly notification to complete the questionnaires. Lastly, the following parameters were assessed at time of a hospital visit, when appropriate: nasal polyp score (NPS: 0–4 on every side, total score 0–8), Lund‐Mackay score (0–24), blood eosinophil counts (BEC), and serum total IgE level. For the current manuscript, a cross‐sectional data analysis was performed with the index date being the day patients were onboarded on the mobile application in the hospital.

### 2.4 Statistics

Descriptive statistics were applied to analyze the collected data. Data are presented as Tukey box–whisker plots. Normality was assessed by Shapiro–Wilk test. Mann–Whitney test was used for between group comparison. Fisher's exact test was used for comparison of proportions between groups. A two‐sided *p* value ≤ 0.05 was considered statistically significant. A non‐parametric ANCOVA was used to compare the patients with BEC ≥ 150 or 250 to the patients with BEC < 150 or 250, while correcting for nasal polyps, asthma, N‐ERD and allergy. In the analysis of the nasal polyp score, we did not adjust for nasal polyps. The results were presented as a relative effect with a 95% confidence interval. Statistical analysis was performed using Graphpad Prism 10 software (Boston, United States).

## 3 Results

### 3.1 Patient Characteristics

This cross‐sectional study included 281 CRS patients (57.9% males–42.1% females) using the Galenus Health app. Mean (± SD) age of the patients was 48.8 (± 12.9) years. Nasal polyps were reported in 82.1% of patients. Type 2 comorbid disease was prevalent with 58.9% reporting a physician diagnosis of asthma, 21.3% of NERD and 57.5% of any type of allergic disease. 68.3% of patients reported a history of ESS and 54.4% reported SCS use in the past year. Detailed patient characteristics are shown in Table 1.

**TABLE 1:** Patient characteristics.

	All patients included	BEC < 150	BEC ≥ 150	Serum total IgE < 100	Serum total IgE ≥ 100	Non‐type 2	Type 2	No asthma	Asthma
# Patients	281	41	149	109	73	29	162	97	139
Age, mean ± SD	48.8 ± 12.9	52.6 ± 12.3	49.1 ± 12.6	49.4 ± 12.9	50.1 ± 12.1	54.7 ± 12.4	48.9 ± 12.3	48.0 ± 12.6	50.1 ± 13.2
Male‐female, %	57.9–42.1	62.5–37.5	56.5–43.5	59.8–40.2	55.6–44.4	60.7–39.3	57.5–42.85	66.0–34.0	51.1–48.9
BMI, mean ± SD	26.2 ± 5.0	25.4 ± 3.9	26.0 ± 4.9	26.1 ± 5.2	25.7 ± 3.9	25.7 ± 4.1	25.9 ± 4.8	25.6 ± 4.0	26.5 ± 5.6
Smoking (curr–ex–non), %	52.5–32.8–14.7	48.1–44.4–7.4	48.9–35.2–15.9	47.5–34.4–18.0	50.0–39.6–10.4	50.0–40.0–10.0	47.9–37.5–14.6	46.5–33.8–19.7	55.6–35.8–8.6
CRSwNP, %	82.8	87.5	91.2	87.9	95.9	82.1	91.9	79.4	89.2
Asthma, %	58.9	52.5	62.0	56.3	64.8	53.6	60.6	0.0	100.0
NERD, %	21.3	27.5	25.5	24.3	28.6	25.0	25.9	8.3	34.4
Allergy, %	57.5	59.0	59.2	52.8	69.4	51.9	60.6	53.8	61.9
Intranasal CS, %	55.4	63.3	46.8	54.2	42.3	68.2	46.7	51.2	54.4
Inhaled CS, %	34.7	30.0	39.6	30.1	48.1	18.2	41.7	13.1	56.3
Systemic CS, %	6.8	6.7	6.3	10.8	0.0	9.1	5.8	2.4	7.8
Prior ESS, %	68.3	82.5	74.3	77.6	75.3	75.0	76.4	60.8	79.9
Prior ESS (0–1–2–3–> 3), %	29.4–43.1–13.3–8.1–6.0	15.4–53.8–12.8–10.3–7.7	30.8–37.7–14.4–8.9–8.2	25.7–48.6–9.5–7.6–6.0	28.8–30.1–20.5–12.3–8.2	18.5–55.6–7.4–11.1–7.4	28.9–39.0–15.1–8.8–8.2	35.6–41.1–13.3–7.8–2.2	21.5–46.2–13.1–10.0–9.2
SCS past year, %	54.4	52.6	59.6	56.6	62.9	59.3	58.2	47.3	58.9
SCS courses past year (0–1–2–3–> 3), %	47.9–17.8–16.5–7.0–10.7	50.0–16.7–25.0–2.8–5.6	42.3–21.1–15.5–7.7–13.4	44.7–22.3–15.5–5.8–10.7	39.7–17.6–22.1–8.8–11.8	44.0–20.0–24.0–4.0–8.0	43.5–20.1–16.9–7.1–12.3	55.6–11.1–21.1–6.7–5.6	42.9–21.4–15.1–6.3–14.3
NPS (0/2–3/4–5/6–7/8)	49.0–27.8–19.6–3.6–0.0	56.4–30.8–7.7–5.1–0.0	45.3–28.1–23.7–2.9–0.0	47.5–27.7–20.8–4.0–0.0	48.7–29.0–20.3–2.9–0.0	59.3–25.9–7.4–7.4–0.0	46.1–28.9–22.4–2.6–0.0	53.6–27.5–15.9–2.9–0.0	46.2–29.9–20.5–3.4–0.0
BEC, median (IQR)	340.0 (200.0–600.0)	100.0 (20.0–100.0)	480.0 (300.0–700.0)	300.0 (198.0–600.0)	400.0 (200.0–600.0)	100.0 (50.0–105.0)	405.0 (250.0–602.5)	300.0 (146.0–500.0)	400.0 (200.0–640.0)
Serum total IgE, median (IQR)	59.0 (10.0–187.0)	28.0 (0.0–148.3)	77.0 (16.7–187.5)	18.6 (0.0–50.5)	228.0 (146.5–346.8)	0.0 (0.0–32.9)	89.2 (18.8–216.0)	38.0 (0.0–147.0)	79.5 (22.8–192.3)

*Note:* Type 2 inflammation is defined as increased blood eosinophil counts (BEC: ≥ 150 cells/μL) and/or serum total IgE (≥ 100 IU/mL).

Abbreviations: BMI, body mass index; CRSwNP, chronic rhinosinusitis with nasal polyps; ESS, endoscopic sinus surgery; NERD, non‐steroidal anti‐inflammatory drug (NSAID)‐exacerbated respiratory disease; NPS, nasal polyp score; SCS, systemic corticosteroids.

### 3.2 Markers of Type 2 Inflammation

CRS patients were stratified according to markers of type 2 inflammation (Table 1): BEC (< 150 cells/μL: 21.6% of patients, ≥ 150 cells/μL: 78.4%; < 250 cells/μL: 36.3%, ≥ 250 cells/μL: 63.7%), serum total IgE (< 100 IU/mL: 59.9%, ≥ 100 IU/mL: 40.1%). According to EPOS, evidence of type 2 inflammation is based on the presence of either increased BEC or serum total IgE. A total of 84.8% of patients fulfill this criterion with a BEC cutoff of 150 cells/μL and 76.4% if a BEC cutoff of 250 cells/μL is applied. In the absence of blood biomarkers, asthma has been proven to be a useful surrogate marker for the presence of type 2 inflammation [30]. Systemic signs of type 2 inflammation were found in 86.2% of patients with asthma. In addition, loss of smell has also been linked with the presence of eosinophils and could therefore potentially serve as surrogate marker of type 2 inflammation [31, 32, 33]. VAS loss of smell ≥ 52 mm, suggested as cutoff for olfactory dysfunction in CRSwNP [34, 35], was observed in 47.6% (123/258) of CRS patients (Table S1).

**FIGURE 1:**
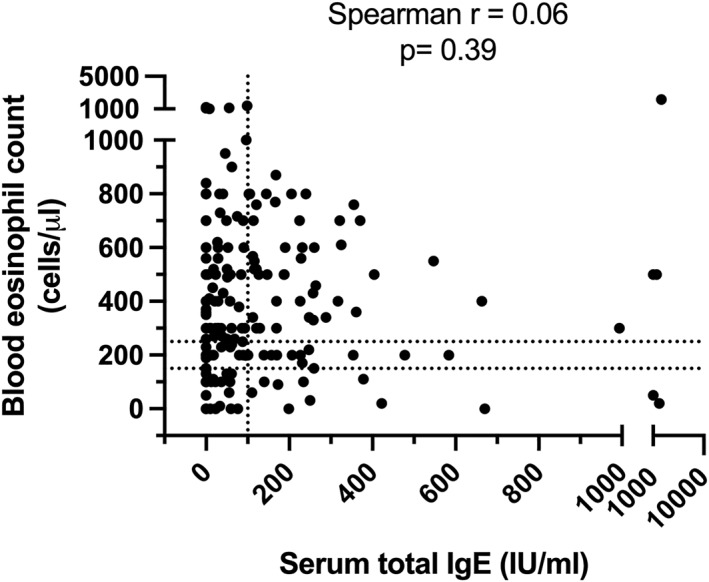
Relationship between serum total IgE and blood eosinophil counts in CRS patients. Spearman test was performed to evaluate the association between both parameters.

Importantly, no significant correlation was shown between BEC and serum total IgE levels (Spearman *r* = 0.06, *p* = 0.39; Figure 1). 48.1% of patients showed both increased BEC (≥ 150 cells/μL) and serum total IgE levels (≥ 100 IU/mL), whereas 16.3% and 21.2% of patients showed only increased BEC or serum total IgE levels, respectively. Additionally, 14.4% of patients showed BEC < 150 cells/μL and serum total IgE levels < 100 IU/mL.

### 3.3 Characteristics of CRS Patients Stratified According to Markers of Type 2 Inflammation

**FIGURE 2:**
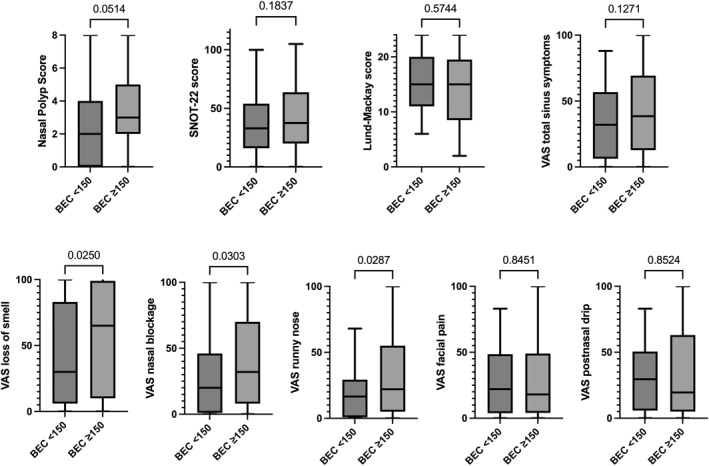
CRS outcomes in patients with and without increased blood eosinophil counts (≥ 150 cells/μL). Data are presented as Tukey box and whisker plots. Mann–Whitney test was performed for between‐group comparison. BEC, blood eosinophil counts; SNOT‐22, sinonasal outcome test‐22; VAS, visual analog scale.

CRS patients with increased BEC, ≥ 150 cells/μL or ≥ 250 cells/μL, respectively, showed higher NPS (*p* = 0.05; *p* = 0.004), SNOT‐22 scores (*p* = 0.18; *p* = 0.03), VAS total CRS symptom scores (*p* = 0.13; *p* = 0.006), VAS loss of smell scores (*p* = 0.03; *p* = 0.04), VAS nasal blockage scores (*p* = 0.03; *p* = 0.004), VAS runny nose scores (*p* = 0.03; *p* = 0.0004) compared to patients with BEC < 150, or < 250 cells/μL (Figure 2 and Figure S1). No significant differences were observed between the high BEC and low BEC groups on LM scores (*p* = 0.57, *p* = 0.34), VAS facial pain scores (*p* = 0.845, *p* = 0.297), VAS postnasal drip scores (*p* = 0.85, *p* = 0.50).

**FIGURE 3:**
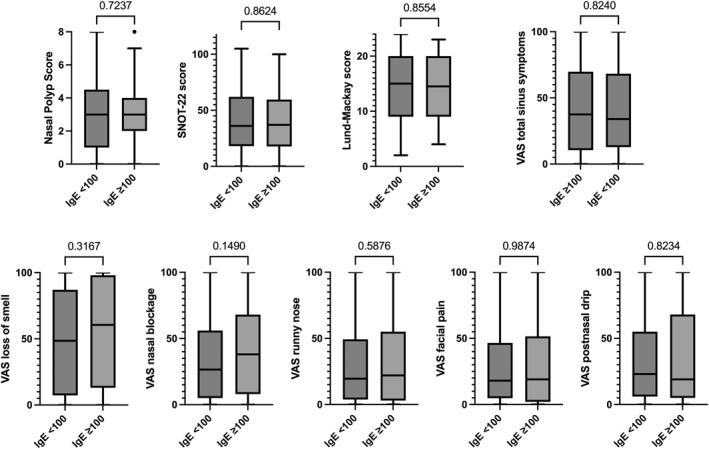
CRS outcomes in patients with and without increased serum total IgE levels (≥ 100 IU/mL). Data are presented as Tukey box and whisker plots. Mann–Whitney test was performed for between‐group comparison. IgE, Immunoglobulin E; SNOT‐22, sinonasal outcome test‐22; VAS, visual analog scale.

CRS patients with increased serum total IgE (≥ 100 IU/mL) did not show differences in any of the clinical or patient‐reported outcome parameters compared to those with serum total IgE < 100 IU/mL (Figure 3).

CRS patients that met the EPOS definition of type 2 inflammation (BEC ≥ 150 cells/μL or serum total IgE ≥ 100 IU/mL), showed increased SNOT‐22 scores (*p* = 0.04), VAS loss of smell scores (*p* = 0.03), VAS nasal blockage scores (*p* = 0.008) and VAS runny nose scores (*p* = 0.005) compared to patients that did not meet this definition (Figure S2). No significant differences were observed between the two groups for NPS (*p* = 0.07), LM scores (*p* = 0.61), VAS total CRS symptom scores (*p* = 0.10), VAS facial pain scores (*p* = 0.43), and VAS postnasal drip scores (*p* = 0.42).

**FIGURE 4:**
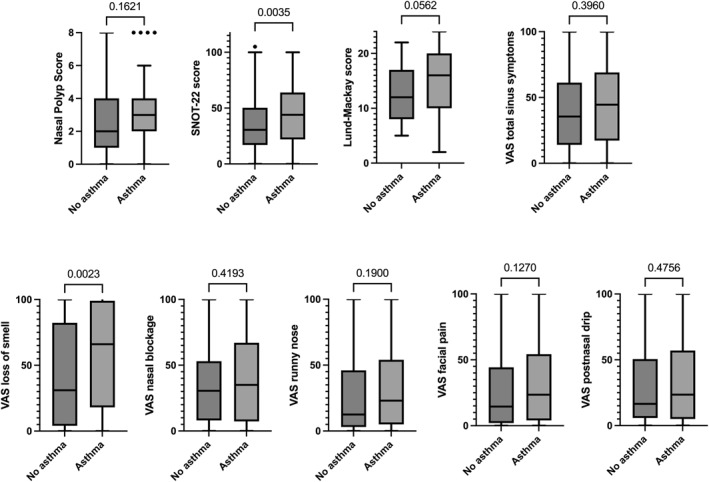
CRS outcomes in patients with and without comorbid asthma. Data are presented as Tukey box and whisker plots. Mann–Whitney test was performed for between‐group comparison. SNOT‐22, sinonasal outcome test‐22; VAS, visual analog scale.

CRS patients with comorbid asthma showed increased SNOT‐22 score (*p* = 0.004) and VAS loss of smell scores (*p* = 0.002) compared to patients without asthma (Figure 4). No differences were observed for the other outcome parameters evaluated (Figure 4). BEC and serum total IgE levels were slightly increased in patients with asthma compared to those without asthma (*p* = 0.05 and *p* = 0.046).

CRS patients with olfactory dysfunction (VAS loss of smell ≥ 52 mm), showed increased NPS (*p* < 0.0001), SNOT‐22 (*p* = 0.0002), BEC (*p* = 0.01) but not serum total IgE (*p* = 0.38; Figure S3).

**FIGURE 5:**
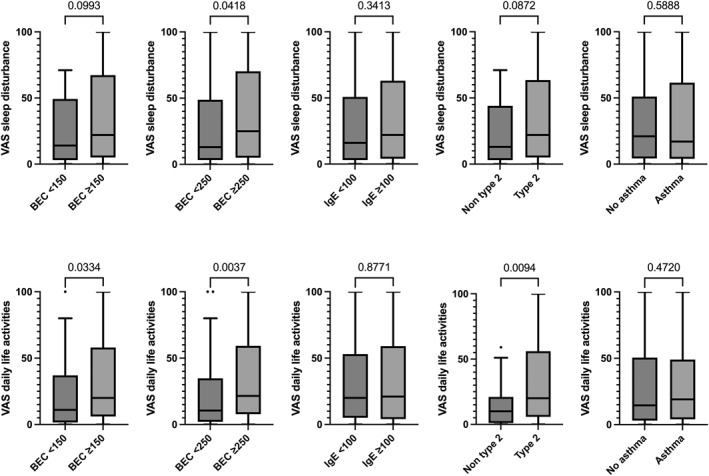
Impact of CRS on sleep disturbance and daily life activities in CRS patients with and without increased markers of type 2 inflammation. Data are presented as Tukey box and whisker plots. Mann–Whitney test was performed for between‐group comparison. BEC, blood eosinophil counts; IgE, Immunoglobulin E; SNOT‐22, sinonasal outcome test‐22; VAS, visual analog scale.

We also evaluated a potential link between markers of type 2 inflammation and either sleep disturbance or daily life activities. Patients with increased BEC (≥ 250 cells/μL), showed increased sleep disturbance compared to patients with normal BEC (*p* = 0.04), whereas patients with increased serum total IgE or with comorbid asthma did not show differences in the level of sleep disturbance compared to patients with serum total IgE < 100 IU/mL or without comorbid asthma (*p* = 0.34 and *p* = 0.59; Figure 5).

CRS patients with increased BEC, ≥ 150 cells/μL or ≥ 250 cells/μL, showed increased VAS impact on daily life activities (*p* = 0.03; *p* = 004), whereas patients with increased serum total IgE or with comorbid asthma did not show differences in VAS impact on daily life activities compared to patients with serum total IgE < 100 IU/mL or without comorbid asthma (*p* = 0.88 and *p* = 0.47; Figure 5).

Lastly, we evaluated the presence of self‐reported asthma, NERD or allergy in patients with high versus low levels of type 2 markers (Table 1). No differences were observed for any of the comorbidities in patients with elevated (either 150 or 250 cutoff) or low levels of BEC, serum total IgE or EPOS definition of type 2 inflammation, except for allergy which was more common in patients with elevated serum total IgE (*p* = 0.03). Patients with comorbid asthma showed a higher proportion of patients with NERD (*p* < 0.0001), but not allergy (*p* = 0.23).

### 3.4 Multivariate Analysis

Patients with BEC ≥ 150 cells/μL showed a significant positive association with VAS LoS (relative effect estimate [95% CI]: 0.60 [0.50–0.70]; *p* = 0.04), VAS NB (0.62 [0.52–0.71]; *p* = 0.02) and VAS runny nose (0.63 [0.53–0.72]; *p* = 0.01) in the multivariate model adjusted for nasal polyps, asthma, N‐ERD and allergy (Table 2). Similarly, patients with BEC ≥ 250 cells/μL showed a significant positive association with NPS (0.61 [0.53–0.70]; *p* = 0.01), SNOT‐22 (0.61 [0.53–0.70]; *p* = 0.01), VAS NB (0.64 [0.58–0.72]; *p* = 0.001) and VAS runny nose (0.67 [0.58–0.75]; *p* = 0.0002; Table 2) in the multivariate model.

**TABLE 2:** Multivariate analysis.

Outcome	BEC 150 cells/μL		BEC 250 cells/μL	
	Relative effect estimate [95% CI]	*p* value	Relative effect estimate [95% CI]	*p* value
Nasal polyp score	0.586 [0.484, 0.688]	0.0970	0.612 [0.528, 0.696]	0.0096
SNOT‐22 score	0.566 [0.473, 0.660]	0.1621	0.607 [0.523, 0.691]	0.0131
VAS loss of smell	0.601 [0.503, 0.698]	0.0431	0.573 [0.489, 0.657]	0.0890
VAS nasal blockage	0.615 [0.518, 0.711]	0.0206	0.640 [0.557, 0.723]	0.0010
VAS runny nose	0.625 [0.526, 0.724]	0.0143	0.668 [0.583, 0.754]	0.0002

*Note:* Relative effect obtained from a nonparametric ANCOVA of the outcome, adjusted for Nasal Polyps, Asthma, Hypersensitivity to NSAIDS and Allergy. The relative effect can be interpreted as the probability of observing a higher response in the BEC ≥ 150 or ≥ 250 group than in the BEC < 150 or < 250 group. 95% CI = 95% confidence interval.

## 4 Discussion

The classification of CRS subtypes has significantly changed in the most recent EPOS 2020 guidelines. Instead of CRSsNP and CRSwNP as major phenotypes, CRS subtypes are currently based on anatomical distribution and endotype dominance [4, 36]. The shift to an endotype‐driven treatment approach [11] has become increasingly important with the advent of biologics as a treatment option for CRS patients with bilateral nasal polyps and underlying type 2 inflammation. In patients with CRSwNP, the presence of blood and tissue eosinophilia, elevated IgE levels, and comorbid asthma are strongly indicative of a type 2 disease, with relevant prognostic and therapeutic implications [37]. Notably, patients with asthma and CRSwNP frequently exhibit a more pronounced type 2 endotype, characterized by the frequent recurrence of nasal polyps and a greater clinical severity [14].

Measurement of tissue eosinophils in the sinonasal mucosa is the preferred (standard) analytical method to determine whether a patient is diagnosed with type 2 disease (tissue eosinophils ≥ 10/high power field) or not. There is ample evidence that eosinophilic infiltration in the sinonasal mucosa correlates with type 2 CRS phenotype and a worse prognosis [38, 39]. Literature data from several studies showed that tissue eosinophil count is a predictor of disease recurrence and poor response to ESS [40, 41]. However, tissue eosinophils are not routinely measured in real‐world settings. Other markers of type 2 inflammation are more frequently used, including analysis of BEC, serum total IgE or the presence of comorbid asthma or subjective smell loss. In the present real‐world study, we showed the lack of correlation between BEC and serum total IgE. From a mechanistic point of view this is not surprising since they are part of different immunological pathways [11]. Differentiation, maturation and activation of eosinophils are dependent on interleukin‐5 (IL‐5), whereas class switching to IgE is dependent on IL‐4 and IL‐13. Importantly, in patients with CRSwNP, IgE is often associated with the presence of superantigens, such as *Staphylococcus* enterotoxin A or B [42], rather than related to a true allergy [43]. Hence, given that BEC and serum total IgE often do not coincide, it is advisable to measure both to have a clear picture of the (sub)endotype in patients with uncontrolled primary CRS.

Measurement of BEC has since long been known as a relevant marker for endotyping in CRS and beyond [44] such as in severe asthma, where it is one of the main clinically applicable biomarkers used in disease management [45, 46]. In the current study, we evaluated the impact of the choice between different BEC cutoff values (150 or 250 cells/μL) on NPS, SNOT‐22, and VAS registered values. The presence of the two cutoff values in the literature results from a change in the hypothetical characteristics of type 2 inflammation between the EPOS2020 guidelines and the following EPOS/EUFOREA 2023 update [4, 47], where the cutoff for BEC was reduced to ≥ 150 in the latter compared to the ≥ 250 of the 2020 guidelines. Our data show that exceeding both cutoff values (150 and 250 cells/μL) is significantly associated with an increase in NPS. Unsurprisingly, this association is more pronounced for the 250 cells/μL cutoff value, suggesting that higher levels of blood eosinophils are associated with more severe disease in terms of sinonasal involvement. The relationship between elevated BEC and SNOT‐22 was less clear but still indicative of a poorer quality of life in patients with elevated BEC. Finally, an increase in BEC correlated with worse symptom scores, as measured by VAS, particularly for loss of smell, nasal obstruction, and runny nose. These findings align with existing literature showing that BEC is not only a marker of type 2 inflammation [45] but, in general, also a marker of disease severity in patients with asthma [48].

In addition to eosinophils, serum total IgE has been used as a determinant in the subtyping of CRS. Previous evidence indicated that IgE plays a central role in amplifying the type 2 inflammatory response in chronic airway disease including CRS [43]. Hence, increased serum total IgE may contribute to the persistence of the type 2 response and promote both symptoms and disease progression [49]. In our study, a 100 IU/mL cutoff for serum total IgE did not show a significant correlation with NPS, SNOT‐22, nor VAS, suggesting that levels above this threshold do not necessarily identify patients with more severe disease if used as a sole marker. However, applying the definition of type 2 inflammation according to EPOS/EUFOREA 2023, considering BEC ≥ 150 cells/μL or IgE ≥ 100 IU/mL, patients with evidence of type 2 inflammation showed increased SNOT‐22 as well as increased VAS for nasal obstruction and runny nose. Taken together, our results cannot confirm the usefulness of serum total IgE as a biomarker of type 2 inflammation in patients with CRSwNP. BEC seem to be the dominant biomarker of type 2 inflammation as recently highlighted by van der Lans et al. [50].

Asthma is often associated with CRSwNP reflecting a more complex phenotype with a prominent type 2 component and more severe (and difficult to control) disease [14]. Our study assessed the presence of comorbid asthma in CRSwNP patients, showing that this comorbidity correlates with increased severity of the SNOT‐22 score and with more pronounced loss of smell. This data suggests that coexistent asthma may worsen clinical manifestations, likely due to an intensified type 2 response activation. This evidence supports the pathophysiological link between the upper and lower airways, with type 2 inflammation as a common denominator, emphasizing the need to consider asthma as a key factor in the phenotypic definition of CRS rather than just comorbidity [13]. Our data support that in clinical practice, the presence of comorbid asthma in patients with CRSwNP could serve as a surrogate marker of type 2 inflammation in the absence of blood biomarkers [30].

In addition to comorbid asthma, loss of smell is also indicative of the presence of type 2 inflammation. Indeed, the presence of eosinophils in the superior turbinate correlated with olfactory dysfunction [33]. Research also showed that the olfactory epithelium might be affected by type 2 inflammatory mediators and in particular basal cells [32]. The subjective scoring of loss of smell by VAS may be useful as a quick tool to identify patients with olfactory dysfunction [34, 35]. In our study, we showed increased disease severity, impact on quality of life and blood eosinophils in patients with increased self‐reported loss of smell.

Our study did not provide data on the level of tissue eosinophils, limiting the possibility of correlating tissue eosinophilia with clinical severity or therapeutic response and is a limitation of this study. Also, it should be acknowledged that our database did not allow for the exclusion of false low BECs due to SCS or type 2 targeting biologic treatment, therefore we cannot exclude that some patients with low BEC are, in fact, patients with suppressed type 2 inflammation. Furthermore, other limitations of the study include the fact that patients were recruited at tertiary referral hospitals with a high proportion of CRSwNP and an underrepresentation of non‐type 2 patients. The high proportion of CRSwNP in this cohort may also be attributed to patient recruitment coinciding with the introduction of biologics in routine clinical practice. Lastly, not all data were available in all patients, which is inherent to the real‐world nature of the study.

In conclusion, defining the type 2 CRS endotype is crucial for the early identification of patients with more severe disease and who may benefit from type 2 targeted biologic therapies. As reported in the EUFOREA2021 manuscript, a significant proportion of CRSwNP patients exhibits a type 2 endotype, characterized by blood eosinophilia, increased serum total IgE and/or concomitant asthma. However, whereas blood eosinophils correlated with symptom severity, the utility of serum total IgE as a biomarker of type 2 inflammation has been shown to be limited based on our findings. Identifying the severe population in CRSwNP remains of importance, as it represents a subgroup with a higher symptom burden, frequent recurrence after endoscopic surgery, and higher impact on quality of life. This facilitates a personalized therapeutic approach with targeted treatment options, aimed at reducing unnecessary surgery, improving patient quality of life, and optimizing the use of health care resources.

## Author Contributions

**Carlo Cavaliere:** writing – original draft, investigation, writing – review and editing. **Sven F. Seys:** conceptualization, funding acquisition, writing – original draft, writing – review and editing, formal analysis, supervision. **Joost de Kinderen:** formal analysis, writing – review and editing. **Giulia Bettio:** formal analysis, writing – review and editing. **Alexandros Andrianakis:** writing – review and editing, investigation. **Isam Alobid:** investigation, writing – review and editing. **Peter W. Hellings:** investigation, writing – review and editing. **Laura Van Gerven:** investigation, writing – review and editing. **Valérie Hox:** investigation, writing – review and editing. **Claire Hopkins:** investigation, writing – review and editing. **Anette Kjeldsen:** investigation, writing – review and editing. **Sietze Reitsma:** investigation, writing – review and editing. **Sven Schneider:** investigation, writing – review and editing. **Peter‐Valentin Tomazic:** investigation. **Zuzana Diamant:** writing – review and editing, supervision. **Julia Eckl‐Dorna:** writing – review and editing, supervision. **Wytske J. Fokkens:** writing – review and editing, investigation. **Clemens Holzmeister:** investigation, writing – review and editing. **Kenneth Larsen:** investigation, writing – review and editing. **Anu Laulajainen‐Hongisto:** writing – review and editing. **Antonella Loperfido:** investigation, writing – review and editing. **Valerie Lund:** writing – review and editing. **Gert Mariën:** conceptualization, funding acquisition, writing – review and editing. **Simonetta Masieri:** investigation, writing – review and editing. **Geoffrey Mortuaire:** investigation, writing – review and editing. **Mathilde Moyaert:** investigation, writing – review and editing. **Joaquim Mullol:** writing – review and editing. **Josje Otten:** investigation, writing – review and editing. **Camilo Rodriquez‐Van Strahlen:** investigation, writing – review and editing. **Martin Wagenmann:** investigation, writing – review and editing. **Claus Bachert:** conceptualization, investigation, funding acquisition, writing – review and editing, supervision.

## Conflicts of Interest

The authors declare no conflicts of interest.

## Supporting information


Supporting Information S1

**Figure S1**: CRS outcomes in patients with and without increased blood eosinophil counts (≥ 250 cells/μL). Data are presented as Tukey box and whisker plots. Mann–Whitney test was performed for between‐group comparison. BEC, blood eosinophil counts; SNOT‐22, sinonasal outcome test‐22; VAS, visual analog scale.

**Figure S2**: CRS outcomes in patients with and without type 2 inflammation. EPOS definition of type 2 inflammation was applied (BEC ≥ 150 cells/μL or serum total IgE ≥ 100 IU/mL). Data are presented as Tukey box and whisker plots. Mann–Whitney test was performed for between‐group comparison. BEC, blood eosinophil counts; SNOT‐22, sinonasal outcome test‐22; VAS, visual analog scale.

**Figure S3**: CRS outcomes and markers of type 2 inflammation in patients stratified by loss of smell (LoS). Data are presented as Tukey box and whisker plots. Mann–Whitney test was performed for between‐group comparison. BEC, blood eosinophil counts; SNOT‐22, sinonasal outcome test‐22; VAS, visual analog scale.

**Table S1**: Characteristics of CRS patients stratified by olfatory dysfunction.

## Data Availability

The data that support the findings of this study are available from the corresponding author upon reasonable request.
