# Supplementary material for: Characterization of Chronic Rhinosinusitis Patients Based on Markers of Type 2 Inflammation: Findings From the European CRS Outcome Registry (CHRINOSOR)
Source: Clin Transl Allergy. 2025 Aug 31;15(9):e70095. doi: 10.1002/clt2.70095 (PMC12399834; doi:10.1002/clt2.70095)
Supplement: Supplementary file 1 — Supporting Information S1 [file CLT2-15-e70095-s001.docx]

**Figure S1. CRS outcomes in patients with and without increased blood eosinophil counts (≥250 cells/μl).**

Data are presented as Tukey box and whisker plots. Mann-Whitney test was performed for between-group comparison. BEC: blood eosinophil counts, SNOT-22: sinonasal outcome test-22, VAS: visual analogue scale.

**Figure S2. CRS outcomes in patients with and without type 2 inflammation.**

EPOS definition of type 2 inflammation was applied (BEC ≥150 cells/µl or serum total IgE ≥100 IU/ml). Data are presented as Tukey box and whisker plots. Mann-Whitney test was performed for between-group comparison. BEC: blood eosinophil counts, SNOT-22: sinonasal outcome test-22, VAS: visual analogue scale.

**Figure S3. CRS outcomes and markers of type 2 inflammation in patients stratified by loss of smell (LoS).**

Data are presented as Tukey box and whisker plots. Mann-Whitney test was performed for between-group comparison. BEC: blood eosinophil counts, SNOT-22: sinonasal outcome test-22, VAS: visual analogue scale.
