# Supplementary figures and images for: Characterization of Chronic Rhinosinusitis Patients Based on Markers of Type 2 Inflammation: Findings From the European CRS Outcome Registry (CHRINOSOR)
Source: Clin Transl Allergy. 2025 Aug 31;15(9):e70095. doi: 10.1002/clt2.70095 (PMC12399834; doi:10.1002/clt2.70095)

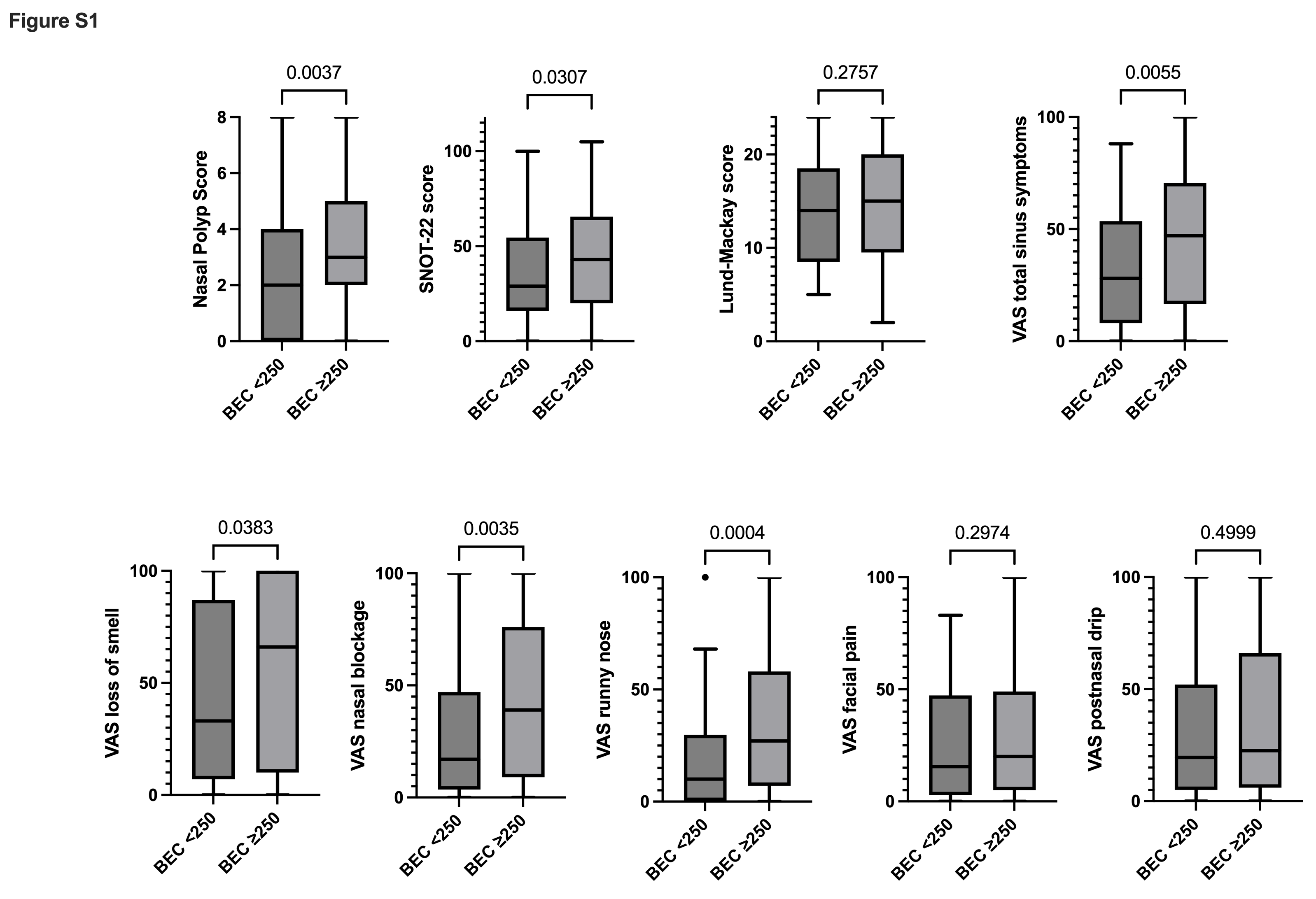

Supplement: Supplementary file 2 — Figure S1: CRS outcomes in patients with and without increased blood eosinophil counts (≥ 250 cells/μL). Data are presented as Tukey box and whisker plots. Mann–Whitney test was performed for between‐group comparison. BEC, blood eosinophil counts; SNOT‐22, sinonasal outcome test‐22; VAS, visual analog scale. [file CLT2-15-e70095-s003.tiff]

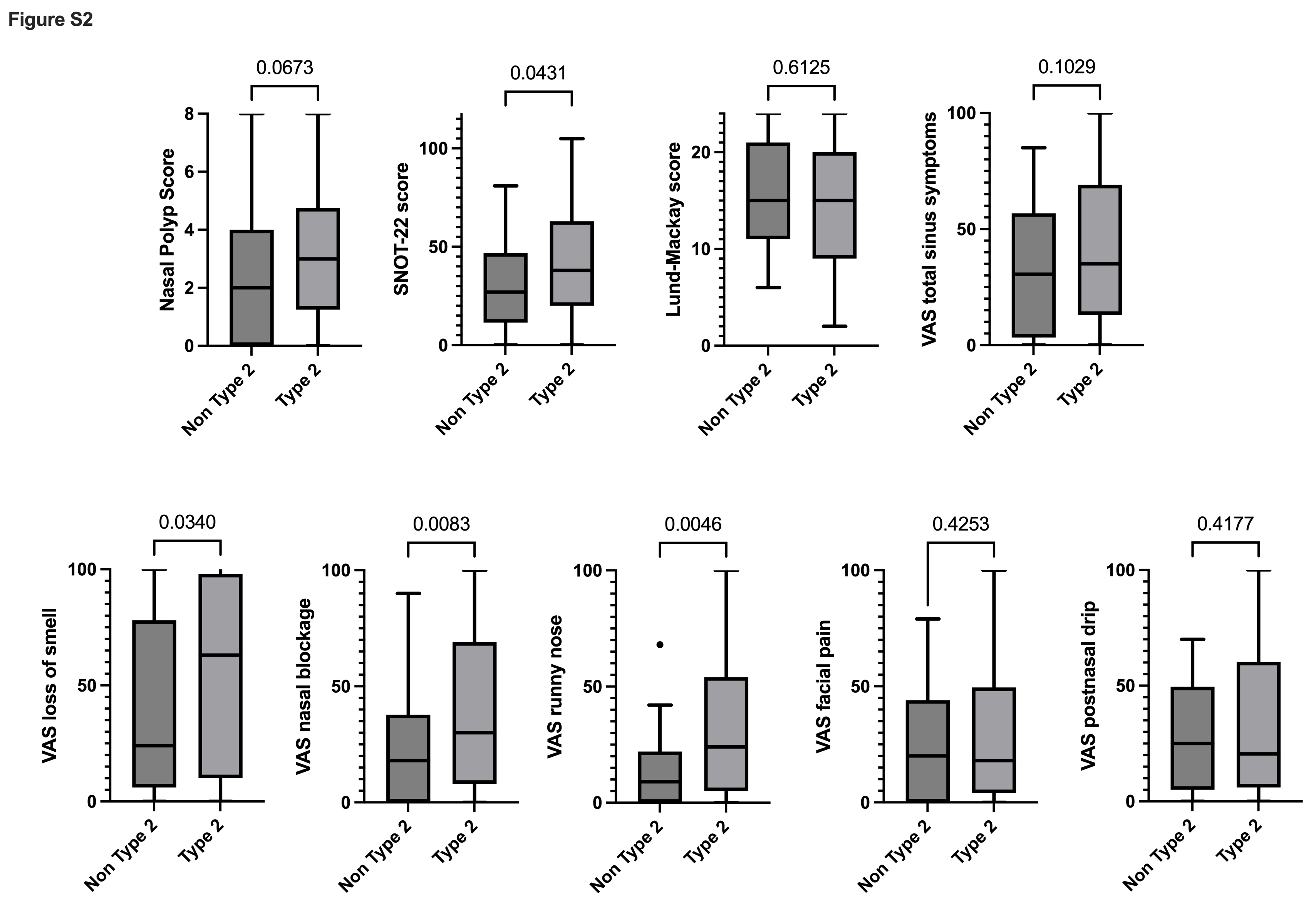

Supplement: Supplementary file 3 — Figure S2: CRS outcomes in patients with and without type 2 inflammation. EPOS definition of type 2 inflammation was applied (BEC ≥ 150 cells/μL or serum total IgE ≥ 100 IU/mL). Data are presented as Tukey box and whisker plots. Mann–Whitney test was performed for between‐group comparison. BEC, blood eosinophil counts; SNOT‐22, sinonasal outcome test‐22; VAS, visual analog scale. [file CLT2-15-e70095-s004.tiff]

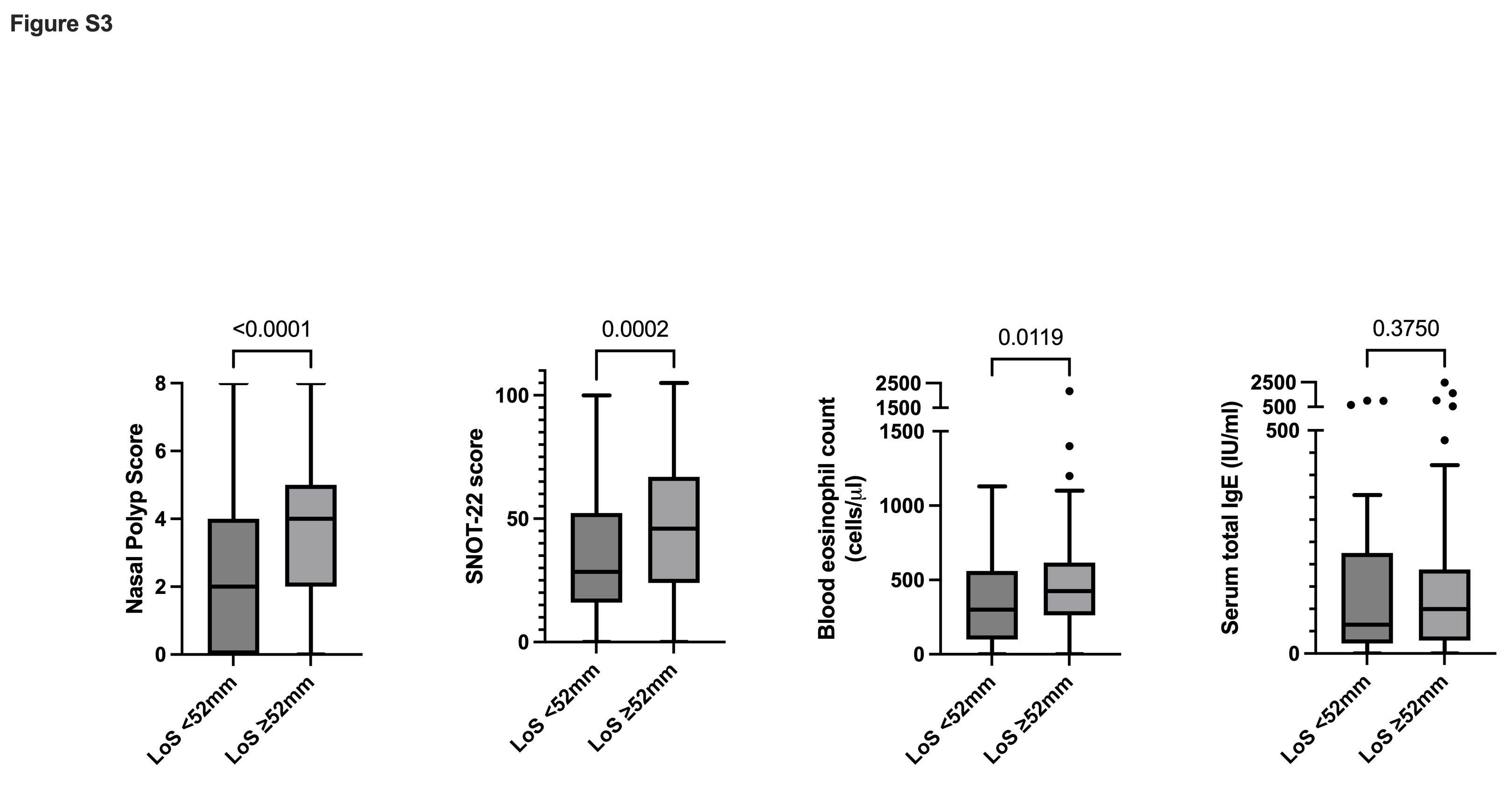

Supplement: Supplementary file 4 — Figure S3: CRS outcomes and markers of type 2 inflammation in patients stratified by loss of smell (LoS). Data are presented as Tukey box and whisker plots. Mann–Whitney test was performed for between‐group comparison. BEC, blood eosinophil counts; SNOT‐22, sinonasal outcome test‐22; VAS, visual analog scale. [file CLT2-15-e70095-s002.tiff]
