# Supplementary material for: Characterization of Chronic Rhinosinusitis Patients Based on Markers of Type 2 Inflammation: Findings From the European CRS Outcome Registry (CHRINOSOR)
Source: Clin Transl Allergy. 2025 Aug 31;15(9):e70095. doi: 10.1002/clt2.70095 (PMC12399834; doi:10.1002/clt2.70095)
Supplement: Supplementary file 5 — Table S1: Characteristics of CRS patients stratified by olfatory dysfunction. [file CLT2-15-e70095-s005.docx]

|  | **VAS LoS <52mm** | **VAS LoS >=52mm** |
| --- | --- | --- |
| **# patients** | 135 | 123 |
| **Age, mean +/-SD** | 47.7 +/- 12.8 | 50.9 +/- 12.3 |
| **Male-female, %** | 57.1 – 42.9 | 58.2 - 41.8 |
| **BMI, mean +/- SD** | 25.9 +/- 4.4 | 26.8 +/- 5.7 |
| **Smoking**  **(curr – ex - non), %** | 54.0 - 31.0 – 14.9 | 48.6 – 35.1 – 16.2 |
| **CRSwNP, %** | 73.1 | 92.7 |
| **Asthma, %** | 51.8 | 67.0 |
| **NERD, %** | 12.4 | 32.5 |
| **Allergy, %** | 58.3 | 56.4 |
| **Prior ESS, %** | 62.4 | 75.2 |
| **OCS past year, %** | 52.9 | 58.6 |
| **Intranasal CS, %** | 59.3 | 53.0 |
| **Inhaled CS, %** | 34.3 | 37.0 |
| **Oral CS, %** | 10.2 | 4.0 |
| **Prior ESS**  **(0 – 1 – 2 – 3 – >3)** | 31.4 – 44.1 – 12.7 – 9.3 – 2.5 | 27.0 - 45.2 - 13.0 – 7.0 - 7.8 |
| **SCS courses past year**  **(0 – 1 – 2 – 3 – >3)** | 48.7 – 14.5 – 19.7 – 5.1 – 12.0 | 44.5 – 22.7 – 15.5 – 8.2 - 9.1 |
| **NPS**  **(0/2 – 3/4 - 5/6 - 7/8)** | 64.5 – 22.6 – 10.8 – 2.2 – 0.0 | 31.1 – 33.3 – 31.1 – 4.4 – 0.0 |
| **BEC, median (IQR)** | 300.0 (100.0 – 560.0) | 425.0 ( 267.5 – 612.5) |
| **Serum total IgE, median (IQR)** | 61.3 (23.0 – 225.0) | 99.8 (29.3 – 183.5) |

**Table S1. Characteristics of CRS patients stratified by olfatory dysfunction.**
